# Flow Cytometry Contributions for the Diagnosis and Immunopathological Characterization of Primary Immunodeficiency Diseases With Immune Dysregulation

**DOI:** 10.3389/fimmu.2019.02742

**Published:** 2019-11-26

**Authors:** Otavio Cabral-Marques, Lena F. Schimke, Edgar Borges de Oliveira, Nadia El Khawanky, Rodrigo Nalio Ramos, Basel K. Al-Ramadi, Gesmar Rodrigues Silva Segundo, Hans D. Ochs, Antonio Condino-Neto

**Affiliations:** ^1^Department of Immunology, Institute of Biomedical Sciences, University of São Paulo, São Paulo, Brazil; ^2^Department of Rheumatology and Clinical Immunology, Faculty of Medicine, Center for Chronic Immunodeficiency (CCI), Medical Center-University of Freiburg, University of Freiburg, Freiburg im Breisgau, Germany; ^3^Immunogenic Inc., São Paulo, Brazil; ^4^Department of Hematology, Oncology and Stem Cell Transplantation, Freiburg University Medical Center, Freiburg im Breisgau, Germany; ^5^Precision Medicine Theme, The South Australian Health and Medical Research Institute (SAHMRI), Adelaide, SA, Australia; ^6^INSERM U932, SiRIC Translational Immunotherapy Team, Institut Curie, Paris Sciences et Lettres Research University, Paris, France; ^7^Department of Medical Microbiology and Immunology, College of Medicine and Health Sciences, UAE University, Al Ain, United Arab Emirates; ^8^Department of Pediatrics, Federal University of Uberlandia Medical School, Uberlândia, Brazil; ^9^Department of Pediatrics, University of Washington School of Medicine, and Seattle Children's Research Institute, Seattle, WA, United States

**Keywords:** flow cytometry, diagnosis, primary immunodeficiency diseases, immune dysregulation, mutation

## Abstract

Almost 70 years after establishing the concept of primary immunodeficiency disorders (PIDs), more than 320 monogenic inborn errors of immunity have been identified thanks to the remarkable contribution of high-throughput genetic screening in the last decade. Approximately 40 of these PIDs present with autoimmune or auto-inflammatory symptoms as the primary clinical manifestation instead of infections. These PIDs are now recognized as diseases of immune dysregulation. Loss-of function mutations in genes such as *FOXP3, CD25, LRBA, IL-10, IL10RA, and IL10RB*, as well as heterozygous gain-of-function mutations in *JAK1* and *STAT3* have been reported as causative of these disorders. Identifying these syndromes has considerably contributed to expanding our knowledge on the mechanisms of immune regulation and tolerance. Although whole exome and whole genome sequencing have been extremely useful in identifying novel causative genes underlying new phenotypes, these approaches are time-consuming and expensive. Patients with monogenic syndromes associated with autoimmunity require faster diagnostic tools to delineate therapeutic strategies and avoid organ damage. Since these PIDs present with severe life-threatening phenotypes, the need for a precise diagnosis in order to initiate appropriate patient management is necessary. More traditional approaches such as flow cytometry are therefore a valid option. Here, we review the application of flow cytometry and discuss the relevance of this powerful technique in diagnosing patients with PIDs presenting with immune dysregulation. In addition, flow cytometry represents a fast, robust, and sensitive approach that efficiently uncovers new immunopathological mechanisms underlying monogenic PIDs.

## Introduction

An effective immune response is required for defending the host from infections as well as playing a fundamental role in physiological homeostasis (1–9). In this context, the investigation of inborn errors of immunity leading to primary immunodeficiency diseases (PIDs) has considerably expanded our understanding of how the immune system works to eliminate infections while avoiding autoimmune diseases (10–17). The first PID was identified in 1952 by Ogden Bruton who reported a male patient with agammaglobulinemia who suffered from recurrent bacterial infections (18). By 2003, mutations in approximately 100 genes were found to cause molecularly defined PIDs (19). The introduction of next-generation sequencing (NGS) (e.g., whole exome sequencing or WES; whole genome sequencing or WGS) led to the discovery of ~120 new genes by 2015 (20–23). The most recent International Union of Immunological Societies (IUIS) report lists more than 320 monogenic causes of PID (24).

**Figure 1:**
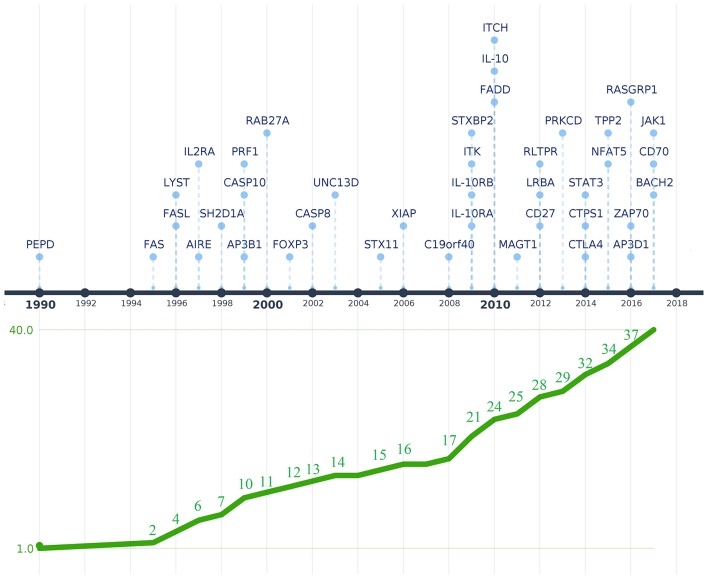
Timeline depicting the discovery of genetic defects that cause PIDs associated with immune dysregulation. Genes are shown above the timeline by year of first reported mutation. The graphic below the timeline shows the cumulative increase of PIDs with immune dysregulation that were genetically characterized. The Y-axis represents the 40 genes associated with diseases of immune dysregulation that were discovered between 1990 and 2017 when the last IUIS phenotypic classification for PIDs was published (shown in the X-axis). The image was created using Time.Graphics (30).

The longitudinal observation and molecular evaluation of PID patients revealed that the phenotype of PID patients comprises not only the susceptibility to bacterial, fungal, and viral infections diseases, but also autoinflammatory and autoimmune disorders as well as an increased incidence of malignancies (15, 16, 25–28). The group of PIDs associated with inflammation and autoimmunity has been recognized by the IUIS Phenotypic Classification Committee for PIDs as “diseases of immune dysregulation” (24). The prototype for this group is the syndrome of Immune Dysregulation, Polyendocrinopathy, Enteropathy, X-linked (IPEX) (29) caused by mutations in the Forkhead Box P3 (*FOXP3*) gene that results in the defective development of CD4^+^CD25^+^ regulatory T cells (Tregs). To date, mutations in some 40 genes have been identified that can present with symptoms of immune dysregulation [Figure 1; (24)]. Patients suspected to have one of these disorders require a rapid and precise diagnosis for prognostic and therapeutic considerations.

Although WES and WGS are powerful tools that have improved the genetic characterization of patients with undefined PIDs, these are laborious, time-consuming, and expensive tests. Flow cytometry, which is readily available in most laboratories, represents a useful low cost and rapid technology for the investigation of PIDs, including patients with symptoms of immune dysregulation. This tool can identify not only the abnormal expression of extra- and intracellular molecules but can also be used to assess functional responses of specific subpopulations of lymphocytes. Flow cytometry-based assays have the advantage of being more quantitative, widely available and relatively easier to perform in a diagnostic laboratory setting compared with other techniques such as western blot analysis, fluorescent and confocal microscopy.

The advantage of using flow cytometry for the diagnosis of PIDs, in general, has been extensively discussed (31–36). Here, we review the progress made in using flow cytometry for the diagnosis of PIDs associated with immune dysregulation and its contributions for a better understanding of disease immunopathology. Although the genetic dissection of several PIDs have provided relevant insights into molecular pathways associated with host defense and immune tolerance (24, 37–43), we discuss here only the inborn errors of immunity presented by the last IUIS phenotypic classification for PIDs in 2017 (44).

### Flow Cytometry for Diseases of Immune Dysregulation

**Figure 2:**
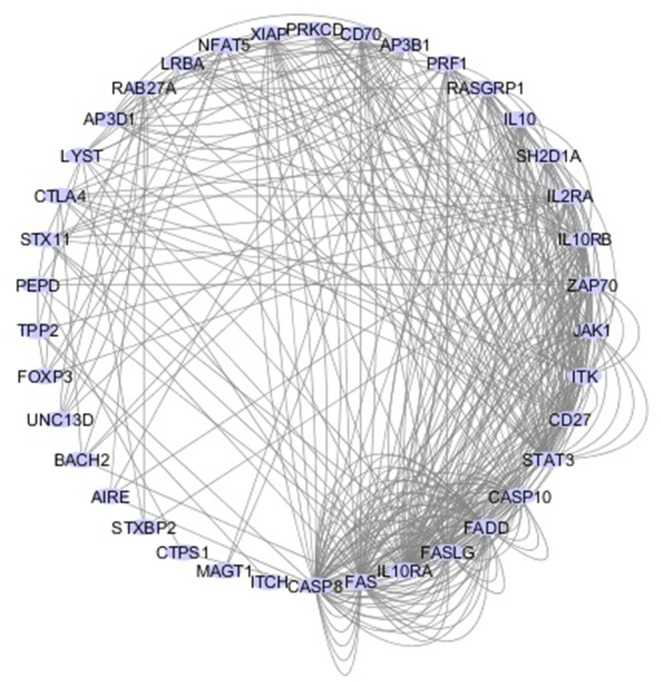
Network of 40 genes that cause PIDs associated with immune dysregulation. The interactive circular graph illustrates the connections (e.g., physical interaction, common signaling pathways, co-localization.) between the causative genes and was developed using the GeneMANIA Cytoscape plugin (47). The genes were provided as a query and are represented by the blue nodes while their connections are represented by the gray lines. Related genes are closer together in the network and have more connecting lines among them.

**Figure 3:**
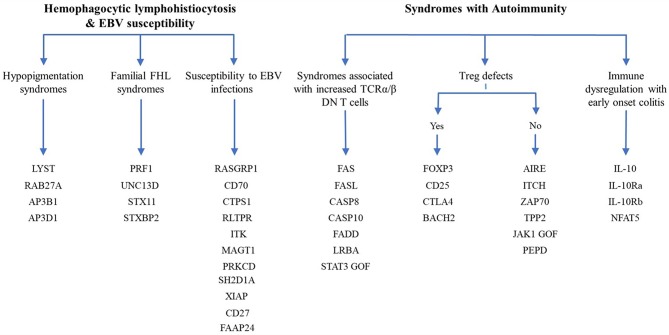
Diagram of the 40 genes that cause PIDs associated with immune dysregulation. The genes are classified according the 2017 IUIS phenotypic classification for PIDs (24).

**Figure 4:**
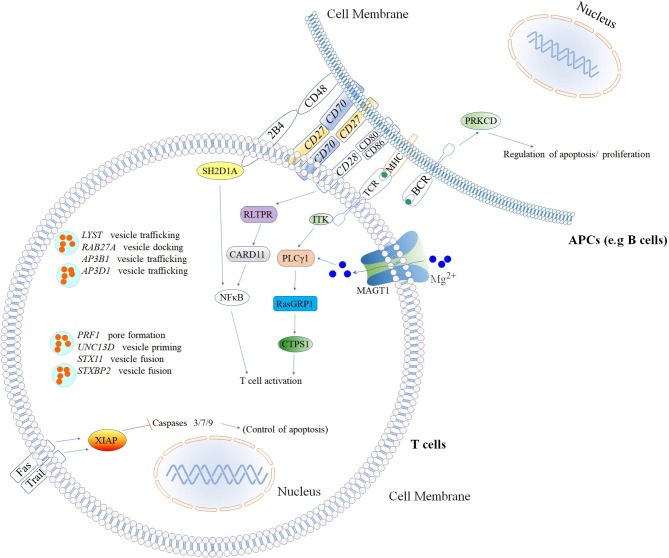
Inborn errors of immunity that cause increased susceptibility to Hemophagocytic Lymphohistiocytosis (HLH) & Epstein–Barr virus (EBV)*. Illustration of mutated genes associated with HLH and increased susceptibility to EBV are shown with colored backgrounds. To allow a better comprehension of signaling pathways involved, other molecules (Fas, Trail, MHC, NFκB, 2BA, CD48, CD28, CD80, CD86, and BCR) not associated with HLH and EBV susceptibility are shown in the white background. *Named according to the 2017 IUIS phenotypic classification for PIDs (24). The illustration was constructed using Motifolio Drawing Toolkits (48).

Since the first attempt by Cooper et al. to provide a classification for PIDs in 1973 (45), the number of PIDs have exponentially increased as most recently summarized by the IUIS Inborn Errors of Immunity Committee classification [Figure 1 (24)]. The first PIDs with features of immune dysregulation appeared in the IUIS Phenotypic Classification for Primary Immunodeficiencies in 1999 (Wiskott-Aldrich syndrome, PNP deficiency, selective IgA deficiency, early complement component deficiencies, and ALPS) (46). In subsequent reports, increased numbers of PIDs with features of immune dysregulation were reported, currently comprising a total of 40 monogenic diseases of immune dysregulation (Figure 2), divided into two main groups labeled “Hemophagocytic Lymphohistiocytosis (HLH) & Epstein-Barr virus (EBV) susceptibility” and “Syndromes with Autoimmunity and Others” (Figure 3). We use this classification throughout this article. The genes causing these disorders are listed in Figure 4 (HLH and EBV susceptibility) and Figure 7 (syndromes with autoimmunity).

With a few exceptions, the flow cytometry contributions for the characterization of diseases of immune dysregulation are discussed below and summarized in Tables 1, 2. We have not included the Fanconi anemia-associated protein 24 (FAAP24) (91) and Itch E3 ubiquitin ligase (ITCH) (92) deficiencies, which are molecules that play a critical role in DNA repair (91, 93) and the negative regulation of T cell activation (92, 94). There are only single reports (91, 92) of these deficiencies and flow cytometry methods for the characterization of their immunopathological mechanisms are not available. This is also the case for prolidase D (*PEPD*) deficiency (95), which has been associated with the development of systemic lupus erythematosus (SLE) (96), and zeta chain of T cell receptor-associated protein kinase 70 (ZAP-70) (97) and nuclear factor of activated T cells 5 (NFAT5) deficiencies (98). Only two ZAP-70-deficient siblings have been reported with combined hypomorphic and activation mutations, and flow cytometry was only used to analyze T cell activation by measuring CD69 expression on activated T cells. Only a single patient with NFAT5 deficiency was reported, for whom flow cytometry was used mainly for immunophenotyping and cell death analysis (98).

**Table 1:** Summary of flow cytometry contributions for the immunopathological characterization of Hemophagocytic Lymphohistiocytosis (HLH) and Epstein–Barr virus (EBV) susceptibility.

**HLH and EBV susceptibility**	**Flow cytometric (FC) application and immunopathological mechanisms identified**	**Genetic defect (References)**	**Inheritance**
Hypopigmentation Syndromes			
Chediak Higashi sd	Reduced degranulation based on the surface up-regulation of CD107a (49) in Natural killer (NK) cells and cytotoxic T lymphocytes (CTLs)	*LYST* (50, 51)	AR
Griscelli sd type 2	Reduced degranulation based on the surface up-regulation of CD107a (49) in NK and CTLs	*RAB27A* (52)	AR
Hermansky-Pudlak sd type 2	Reduced degranulation based on the surface up-regulation of CD107a (49) in NK and CTLs	*AP3B1* (53)	AR
Hermansky-Pudlak sd, type 10	Reduced degranulation based on the surface up-regulation of CD107a (49) in NK and CTLs	*AP3D1* (54)	AR
Familial HLH			
Perforin deficiency (FHL2)	Perforin expression in NK cells and CTLs Normal CD107a expression in NK and CTLs	*PRF1* (55)	AR
UNC13D or Munc13-4 deficiency (FHL3)	Munc13-4 expression in NK cells, CTLs, and platelets.	*UNC13D* (56)	AR
Syntaxin 11 deficiency (FHL4)	STX11 expression not available by FC (no antibody validated). Reduced CD107a expression in NK and CTLs	*STX11* (57)	AR
STXBP2 or Munc18-2 deficiency (FHL5)	STXBP2 expression by FC not available (no antibody validated). Reduced CD107a expression in NK and CTLs	STXBP2 (58)	AR
Susceptibility to EBV infections RASGRP1 deficiency	Reduced cell proliferation using fluorescent cell staining dye; impaired T cell activation by measuring CD69 expression; defective CTPS1 expression; reduced intracellular expression of active caspase 3; reduced T cell apoptosis using annexin V/propidium iodide staining, all in response to CD3/TCR activation	RASGRP1 (59–63)	AR
CD70 deficiency	CD70 expression on phytohaemagglutinin (PHA)-stimulated T cells; binding of a CD27-Fc fusion protein on T cells	CD70 (64)	AR
CTPS1 deficiency	Defective cell proliferation using fluorescent cell staining dye	CTPS1 (65)	AR
RLTPR deficiency	RLTPR expression in adaptive (B and T lymphocytes) and innate (monocytes and dendritic cells) immune cells. Reduced phospho-nuclear factor (NF)-κB P65-(pS259) expression and inhibitor (I)κBα degradation in CD4^+^ and CD8^+^, specifically after CD28 co-stimulation; CD107a expression after K562 stimulation	RLTPR or CARMIL2 (66)	
ITK deficiency	ITK expression by FC not available (no antibody validated). Reduced T cell receptor (TCR)-mediated calcium flux; absence of Natural Killer T (NKT) cells determined as TCR Vβ11 and TCR Vα24 double-positive cells	ITK (67)	AR
MAGT1 deficiency	MAGT1 expression by FC not available (no antibody validated). Reduced CD69 expression in CD4^+^ T cells after anti-CD3 stimulation. Low CD31^+^ cells in the naïve (CD27^+^, CD45RO^−^) CD4^+^ T cell population. Impaired Mg influx using Mg2^+^-specific fluorescent probe MagFluo4. Reduced NKG2D expression in NK cells and CTLs	MAGT1 (68)	XL
PRKCD deficiency	Increased B cell proliferation after anti-IgM stimulation; resistance to PMA-induced cell death; low CD27 expression on B cells	PRKCD (69–71)	AR
XLP1	SH2D1A expression, low numbers of circulating NKT cells (Vα24TCR+/Vβ11TCR+). Impaired apoptosis.	SH2D1A (72)	XL
XLP2	XIAP expression, low numbers of circulating NKT cells (Vα24TCR+/Vβ11TCR+). Enhanced apoptosis	XIAP (73)	XL
CD27 deficiency	CD27 expression on B cells	CD27 (74)	AR

*Diseases are classified as reported by the 2017 IUIS phenotypic classification for PIDs (24). AP3B1, Adaptor Related Protein Complex 3 Beta 1; AP3D1, Adaptor Related Protein Complex 3 Delta 1; AR, Autosomal recessive; CD27, Cluster of Differentiation 27; CD70, Cluster of Differentiation 70; CTPS1, Cytidine triphosphate synthase 1; FHL, familial hemophagocytic lymphohistiocytosis; ITK, IL2 Inducible T Cell Kinase; LYST, Lysosomal Trafficking Regulator; MAGT1, Magnesium Transporter 1; PRF1, Perforin 1; PRKCD, Protein Kinase C Delta; RAB27A, Ras-Related Protein Rab-27A; RASGRP1, RAS guanyl-releasing protein 1; RLTPR, RGD motif, leucine rich repeats, tropomodulin domain and proline-rich containing; sd, syndrome; SH2D1A, SH2 Domain Containing 1A; STX11, Syntaxin 11; STXBP2, Syntaxin Binding Protein 2; UNC13D, Protein unc-13 homolog D; XL, X-linked; XIAP, X-linked inhibitor of apoptosis protein*.

**Table 2:** Summary of flow cytometry contributions for the immunopathological characterization of Syndromes with autoimmunity.

**Syndromes with autoimmunity**	**Flow cytometric (FC) application and immunopathological mechanisms identified**	**Genetic defect (References)**	**Inheritance**
Syndromes associated with increased TCRα/β DN T cells			
ALPS-FAS	FAS expression, reduced T cell apoptosis	*TNFRSF6* (75)	AD/AR
ALPS-FASLG	FASL expression, reduced T cell apoptosis	*TNFSF6* (76)	AD/AR
ALPS-Caspase8	Reduced T cell apoptosis	*CASP8* (77)	AR
ALPS-Caspase 10	Reduced T cell apoptosis	*CASP10* (78)	AD
FADD deficiency	Reduced T cell apoptosis	*FADD* (79)	AR
LRBA deficiency	Reduced T regulatory (T reg) cells, low CTLA4 and Helios; Increased B cell apoptosis and low levels of IgG^+^/IgA^+^ CD27^+^ switched-memory B cells; reduced B proliferative capacity, and impaired activation (using CD138 staining)	LRBA (80)	AR
STAT3 gain-of-function (GOF) mutation	Delayed de-phosphorylation of STAT3; diminished STAT5 and STAT1 phosphorylation; which is in line with the role in the negative regulation of several STATs162. High levels of Th17 cells; reduced FOXP3^+^CD25^+^ Treg population; decreased FASL-induced apoptosis	STAT3 (81)	AD
Defective regulatory T cells			
IPEX	Decreased or absent FOXP3 expression by CD4^+^CD25^+^ regulatory T cells	FOXP3 (82)	XL
CD25 deficiency	Impaired CD25 expression; defective proliferative responses following anti-CD3 or PH; defective NK cell maturation increased (CD56brightCD16hi and reduced CD56dimCD16hi NK cells in peripheral blood); increased degranulation by elevated CD107a expression and higher perforin and granzyme B expression in NK cells;	CD25 or IL2RA (83)	AR
CTLA4 haploinsufficiency	CTLA4 expression, trafficking, binding to its ligand, and CTLA4-mediated trans-endocytosis	CTLA4 (84)	AD
BACH2 deficiency	Reduced BACH2 expression in T and B lymphocytes, decreased FOXP3 expression by CD4^+^CD25^+^ regulatory T cells, reduced total and class-switched memory B cells, increased T-bet expression	BACH2 (85)	AD
Normal regulatory T cell function			
APECED	Expression of IL-17A, IL-17F, and IL-22 by PBMCs. AIRE expression by FC is not available (no antibody validated)	AIRE (86)	AR
Tripeptidyl-Peptidase II deficiency	Lymphocytes expressing high levels of major histocompatibility complex (MHC) class I molecules, a predominant T CD8^+^CD27^−^CD28^−^CD127^−^ phenotype; increased percentage of IFN-γ and IL-17 positive T cells; high expression of T-bet and perforin. Defective proliferation lymphoproliferation and increased susceptibility to apoptosis; increased levels of CD21low B cells	TPP2 (87)	AR
JAK1 GOF	Increased JAK1, STAT1, and STAT3 phosphorylation	JAK1 (88)	AD
Immune dysregulation with early onset Colitis			
IL-10 deficiency	No FC assay available. Normal STAT3 phosphorylation in response to IL-10	IL-10(89)	AR
IL-10RA deficiency	IL-10RA expression; defective STAT3 phosphorylation in response to IL-10. Normal STAT3 phosphorylation in response to IL-23	IL-10Ra (90)	AR
IL-10RB deficiency	IL-10RB expression; defective STAT3 phosphorylation in response to IL-10. Normal STAT3 phosphorylation in response to IL-23	IL-10Rb (90)	AR

*Diseases are classified as reported by the 2017 IUIS phenotypic classification for PIDs (24). AD, Autosomal dominant; ALPS-FAS, Autoimmune lymphoproliferative syndrome-Fas cell surface death receptor; ALPS-FASLG, Autoimmune lymphoproliferative syndrome FAS ligand gene; APECED, Autoimmune polyendocrinopathy candidiasis ectodermal dystrophy; AR, Autosomal recessive; BACH2, BTB Domain And CNC Homolog 2; CASP8, cysteine-aspartic acid protease 8;CASP10, cysteine-aspartic acid protease 10; CD25 or IL2RA, Interleukin 2 Receptor A; CTLA4, cytotoxic T-lymphocyte-associated Protein 4; DN, double negative; FADD, Fas Associated Via Death Domain; IL-10, Interleukin-10; IL-10Ra, Interleukin-10 Receptor alpha; IL-10Rb, Interleukin-10 Receptor beta; IPEX, Immune dysregulation; polyendocrinopathy; enteropathy; XL, X-linked; JAK1, Janus Kinase 1; LRBA, LPS Responsive Beige-Like Anchor Protein; NFAT5, Nuclear Factor Of Activated T Cells 5; STAT3, signal transducer and activator of transcription 3; TPP2, Tripeptidyl Peptidase 2*.

### Flow Cytometry Guidelines

Before reviewing the contribution of flow cytometry to the characterization of PIDs with immune dysregulation, we emphasize that in order to perform molecular characterization of inborn errors of immunity in diagnostic laboratories, one needs to become familiar with the flow cytometry guidelines and parameters, which have been previously reported (31, 99–104) They were discussed in detail with focus on technical flow cytometry aspects. For example, flow cytometry parameters of general importance are the determination and validation of flow cytometry positive controls (e.g., fluorescence compensation controls as well as resting and activation controls in the case of inducible molecules), the establishment of appropriate cutoffs (e.g., by defining the 10th percentile of normal controls as a center-specific lower limit of normal), and avoiding misinterpretation of results due to inter-laboratory variability, specificity, and sensitivity, particularly in patients with low peripheral blood lymphocyte counts. Another important issue is that some functional assays have a time frame (normally within 24 h after venous puncture) within the test must be performed, due to changes in cell viability or the activation of affected cell pathways during blood shipment. Thus, it is important to obtain blood from healthy controls at the same time of patient sampling and ship them together for flow cytometry screening tests (49, 105). In cases that the cells obtained from the same-day healthy control show results outside the normal range, i.e., not expressing or overexpressing a specific molecule, which is used as experimental readout such as in degranulation assays (49), the shipment and test have to be repeated. Altogether, the above mentioned factors as well as other experimental procedures such as correct definition of instrument setup and evaluation of cell viability prior to the experiment are of major importance for the proper execution of diagnostic flow cytometry. Importantly, following the initial flow cytometry screening tests, there is a significant amount of work to be performed by functional validation studies (e.g., by combining site-directed mutagenesis combined with flow cytometric assays) when identifying new molecular defects.

## Hemophagocytic Lymphohistiocytosis and EBV Susceptibility

HLH is a hyper-inflammatory syndrome directly linked to abnormalities in cytotoxicity as a result of defective degranulation. This syndrome is characterized by prolonged fever and massive hepatosplenomegaly associated with laboratory findings such as cytopenia, hypertriglyceridemia, hypofibrinogenemia, and NK cells and cytotoxic (CD8^+^) T lymphocytes (CTLs) exhibiting reduced cytotoxicity (24, 106). Clinical and immunological features of FHL syndromes have previously been reviewed in detail (107, 108). Natural killer (NK) and cytotoxic T cells from these patients show an impaired capacity to control viral infections. The unique curative therapy for HLH is hematopoietic stem cell transplantation (HSCT) (109–111).

**Figure 5:**
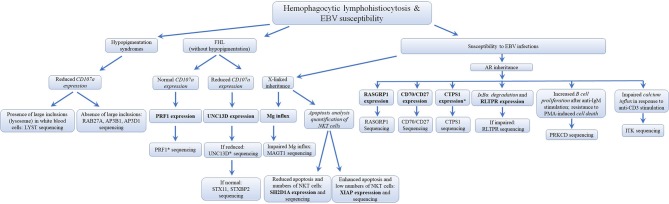
Flowchart depicting the immunophenotypic analysis used to define the molecular genetic defects of patients with hemophagocytic lymphohistiocytosis & EBV susceptibility, with flow cytometry. In those cases with normal protein by flow cytometry, if there is a strong clinical indication for a specific immune dysregulation disease, it is recommended to perform gene sequencing to exclude missense mutations that do not impair protein expression. While it has been estimated that PRF1 deficiency accounts for 30–60% of known FHL cases and UNC13D deficiency for up to 20% of FHL cases, the frequency for most of the other immune dysregulation syndromes remains unknown. *Flow cytometry assay validated in HepG2 cells, but remains to be tested with cells from PID patients. Bold and italic texts are disease-specific and non-disease specific flow cytometry tests, respectively.

Several different genetic disorders are associated with an HLH phenotype and are classified as HLH with hypopigmentation or without hypopigmentation (familial hemophagocytic lymphohistiocytosis syndromes or FHL). Secondary HLH, generally seen in older children and adults without a known genetic defect, are triggered by viral infections such as EBV (most commonly), cytomegalovirus, and herpes simplex virus, or by hematologic malignancies, rheumatologic conditions, or tuberculosis (112). The 19 causative genes associated with the HLH and EBV susceptibility group are summarized in Figure 4 as well as a summarized guideline is shown in Figure 5, which describes the flow cytometric assays required to diagnose patients with syndromes that present with autoimmunity.

### Hypopigmentation Syndromes

Four different inborn errors of immunity causing HLH with hypopigmentation/albinism have been described: Chediak-Higashi syndrome, genetically characterized by mutations in the lysosomal trafficking regulator (*LYST*) gene (50, 51); Griscelli syndrome type 2 due to mutations in Ras-Related Protein Rab-27A (*RAB27A*) (52); and Hermansky-Pudlak syndrome type 2 and type 10 caused by mutations in the adaptor related protein complex 3 beta 1 (*AP3B1*) (53) and adaptor related protein complex 3 delta 1 (*AP3D1)* (54), respectively. These diseases generally manifest as hypopigmentation, immunodeficiency, neutropenia, or decreased NK and cytotoxic T cell activity, and bleeding tendency. However, a few cases of patients with Griscelli syndrome type 2 with biallelic mutations in *RAB27A* have been reported with normal pigmentation (113).

Interestingly, the overlapping clinical features shown by inborn errors of immunity causing HLH with hypopigmentation/albinism might be explained by defects in the molecular machinery responsible for the biogenesis and transport of secretory lysosome-related organelles in different cell types (54). These physiologic processes are essential for production and secretion of perforin and granzyme by NK cells and cytotoxic CD8^+^ T lymphocytes (54, 55), as well as secretion of melanin by melanosomes (114, 115) and release of small molecules by δ granules from platelets during blood vessel damage, which facilitates platelet adhesion and activation during coagulation (114).

Since there is no specific flow cytometry approach established to detect the expression of LYST, RAB27A, AP3B1, or AP3D1, the differential diagnosis of these syndromes, based on flow cytometry, is not possible and thus the diagnosis relies on biochemical and molecular criteria (50, 109, 116). A few specific features differentiate these disorders, such as the presence of large inclusions (lysosome) in white blood cells from patients with Chediak Higashi syndrome (49, 50, 116), specific hair shaft anomalies, and the detection of a platelet storage pool deficiency characteristic of Hermansky-Pudlak syndrome (54). However, flow cytometry has been used successfully as a screening tool for primary (i.e., genetic) degranulation defects. The approach relies on measuring the up-regulation of CD107a on NK cells (with/without K562 stimulation) (49) and cytotoxic T lymphocytes (with/without anti-CD3 stimulation) (54). CD107a is a lysosomal protein that co-localizes with perforin and granzyme in cytolytic granules (117, 118) and is expressed on the cell surface upon activation-induced degranulation following the engagement of T cell receptor (TCR) and NK cell activating receptors (119, 120). This assay has been performed in parallel with a cytotoxicity assay using K562 or P815 target cells to functionally confirm the degranulation defect suggested by a reduced CD107a expression following 48 h with phytohemagglutinin (PHA)/IL-2 or anti-CD3/anti-CD28 beads stimulation (49, 109, 112, 121). This degranulation assay allows the differentiation between primary and secondary HLH. The latter express CD107a normally upon cell activation (49). Furthermore, as elegantly reported by Bryceson et al. (49), the analysis of CD107a expression by flow cytometry has the advantage of being a sensitive assay even when patients receive immunosuppressive therapy or have very low numbers of T/NK cells. Detailed methodological information about the detection of T and NK cell degranulation by flow cytometry can be found elsewhere (36, 122).

### Familial Hemophagocytic Lymphohistiocytosis (FHL) Syndromes

FHL is a life-threatening autosomal-recessive inherited hyper-inflammatory syndrome that usually develops within the first 2 years of age (56). FHL syndromes are caused by mutations in *perforin-1* (*PRF1*), designated as FLH2, accounting for 30–50% of known cases (105, 108), or proteins involved in perforin secretion: protein unc-13 homolog D (*UNC13D*) (56), Syntaxin-11 (*STX11*) (57), and Syntaxin Binding Protein 2 (*STXBP2*) (58), known as FHL3, FHL4, and FHL5, respectively. The gene responsible for FHL1 has not yet been identified (107).

Defective perforin expression by NK cells (CD3^−^CD56^+^CD16^+^) and cytotoxic T lymphocytes (CD3^+^CD8^+^) can be detected by flow cytometry and has been used as a screening approach for FHL2 (34). Likewise, patients with UNC13D deficiency, which accounts for up to 20% of FHL cases, can be identified by decreased UNC13D expression using flow cytometry. Usually, UNC13D expression is assessed on NK cells and T lymphocytes. Since patients with UNC13D deficiency frequently present with significantly reduced leukocyte counts (pancytopenia), UNC13D expression can instead be analyzed on platelets (CD41a^+^) (34, 105, 123), since platelets express UNC13D more abundantly than peripheral blood leukocytes (105).

There is no specific or commercially available antibody for flow cytometry to screen patients with STX11 and STXBP2 deficiencies. Therefore, these two deficiencies have been identified indirectly by measuring CD107a expression, or by the exclusion of defective PRF1 and UNC13D expression. While cells from patients with FHL3-5 present reduced CD107a expression on the surface of NK cells and CTLs, CD107a expression is normal in subjects with PRF-1 deficiency (49, 58, 123). This phenomenon is explained by the fact that perforin constitutes part of the lytic granule content, but in contrast to UNC13D, STX11, and STXBP2, it is not essential for the transport of secretory lysosome-related organelles (55, 58, 106, 107, 124, 125).

### Susceptibility to EBV Infection

More than 90% of the global population are EBV-seropositive, with the majority being asymptomatic or manifesting a self-limiting disease (126). Patients with inborn errors of immunity that result in susceptibility to EBV may develop severe or fatal mononucleosis, B cell lymphoma, lymphoproliferative disease, or HLH (67, 127–129). Mutations in at least 11 genes (four of them with *EBV-associated HLH*) are known to cause increased susceptibility to EBV (24), demonstrating the non-redundant role of signaling pathways that generate EBV-specific immunity, and the pivotal role of continuous immune surveillance to ensure virus-host homeostasis (129, 130). The signaling pathways and outcomes involved in the immunopathogenesis of severe EBV infections (129) are summarized in Figure 4.

Notably, T cell proliferation by patients with susceptibility to EBV can be reduced, normal or even increased (Table 3); however, some subjects belonging to the same PID subgroup may display variable proliferation results where some patients with CTPS1 (65, 131) and CD27 (74, 134) deficiencies have reduced T cell proliferation and others do not. Moreover, the abnormal proliferative responses might be stimulus dependent. For instance, patients with ITK deficiency may demonstrate reduced T cell proliferation in response to CD3/CD28 stimulation, but normal proliferation in response to PHA stimulation (132). Therefore, in addition to be a non-specific assay to screen different PIDs, the analysis of T cell proliferation from patients with susceptibility to EBV needs to be carefully scrutinized as a screening flow cytometry tool to direct the definitive diagnosis of these PIDs.

**Table 3:** T cell proliferation response of PIDs with susceptibility to EBV.

**Susceptibility to EBV**										
Deficiency	RASGRP1	CD70	CTPS1	RLTPR	ITK	MAGT1	PRKCD	XLP1	XIAP	CD27
T cell proliferation	Reduced	Reduced	Reduced	Reduced	Reduced	Reduced	Normal	Increased	Reduced	Reduced
References	(60)	(64)	(65, 131)	(66)	(132)	(68)	(70)	(133)	(73)	(74, 134)

#### RASGRP1 Deficiency

RAS guanyl-releasing protein 1 (*RASGRP1*) is a guanine nucleotide exchange factor and activator of the RAS-MAPK pathway initiated by diacylglycerol following TCR signaling (129). Mutations in RASGRP1 have been found in patients with a combined immunodeficiency (a ALPS-like disease) (59) presenting with recurrent respiratory infections in association with EBV-induced lymphoproliferative disease, chronic lymphadenopathy, hepatosplenomegaly, autoimmune hemolytic anemia, and immune thrombocytopenia (59–63). In addition to its availability as a screening tool to establish the diagnosis of RASGRP1 deficiency (59), flow cytometry has been widely applied to evaluate functional defects resulting from RASGRP1 mutations. For instance, this approach can be used to detect reduced T cell expansion by a cell proliferation kit (e.g., CellTrace), impaired T cell activation by CD69 staining, and markedly reduced phosphorylation of ERK. Diminished intracellular expression of active caspase 3 in lymphocytes associated with reduced apoptosis using annexin V (AV) and propidium iodide (PI) staining has been observed (59–61).

#### CD70/CD27 Deficiencies

Disorders of T cell co-signaling pathways such as those caused by deficiencies in CD40L, SAP, OX40, or CD70/CD27 highlight the critical role of co-stimulation for host defense (135–137). Patients with mutations affecting the co-stimulatory molecules CD70 and CD27 (Figure 4), which are expressed on the surface of T, B and NK cells (138–140) present with similar clinical phenotypes. These patients exhibit impaired effector CD8^+^ T cell generation, hypogammaglobulinemia, lack of memory B cells, and reduced cytolytic and proliferative responses of T cells resulting in chronic EBV infections (EBV-associated lymphoproliferation, EBV-associated HLH, and B cell lymphoma). Additionally, affected patients might develop severe forms of other viral infections including influenza, herpesviruses (e.g., varicella-zoster virus), and cytomegalovirus (CMV) (64, 74, 134, 141–143). Cell-surface expression of both CD70 and CD27 are assessed by flow cytometry using specific monoclonal antibodies. Similar to other combined deficiencies, it is possible that a mutated non-functional protein is expressed on the cell surface (144, 145) in which case it is possible to analyse the ability of a CD27Fc fusion protein that binds to CD70, by flow cytometry (64).

#### RLTPR Deficiency

The RLTPR (RGD motif, leucine-rich repeats, tropomodulin domain, and proline-rich containing) is a scaffold protein that bridges CD28 located on the cell-surface to the cytosolic adaptor called Caspase Recruitment Domain Family Member 11 (CARD11), enabling proper activation of the TCR-induced NF-κB signaling pathway (146, 147). Although human CD28 deficiency has not yet been characterized, RLTPR deficiency was recently reported as an autosomal recessive combined immunodeficiency highlighting the critical role of the CD28 pathway for T- and B-cell activation (66). RLTPR-deficient patients present with low numbers of memory CD4^+^ T cells, reduced numbers of T helper (Th)1, Th17, and T follicular helper cells, as well as reduced memory B cells, and show poor antibody responses to vaccines (67, 148). RLTPR deficiency causes susceptibility to a variety of pathogens, including bacteria, fungi, and viruses (e.g., EBV). RLTPR expression can be detected by flow cytometry in adaptive (B and T lymphocytes) and innate (monocytes and dendritic cells) immune cells. Moreover, NF-κB signaling defects (149, 150) in CD4^+^ and CD8^+^ T cells from patients with RLTPR mutations have been characterized by flow cytometry, primarily manifested by reduced NF-κB P65 phosphorylation and IκBα degradation following anti-CD28 stimulation (66). In this context, there is a debatable paradigm that CD28 co-stimulation is not necessary for the activation of memory T cells. In agreement, flow cytometric analysis of T cell proliferation has shown that the lack of RLTPR only impairs the proliferation of naïve, but not memory T cells (66). Flow cytometric analysis also points out a critical role of RLTPR in NK cells, since their degranulation capacity is impaired after K562 stimulation, depicted by reduced CD107a expression (151).

#### CTPS1 Deficiency

The cytidine nucleotide triphosphate synthase 1 (*CTPS1*) is a molecule involved in DNA synthesis in lymphocytes (152) and therefore plays a central role in lymphocyte proliferation (65, 131). Loss-of-function homozygous mutations in CTPS1 cause a combined immunodeficiency characterized by the impaired capacity of activated T and B cells to proliferate in response to antigen receptor-mediated activation (65). CTPS1-deficient patients are susceptible to life-threatening bacterial and viral infections, including those caused by EBV (e.g., EBV-related B-cell non-Hodgkin lymphoma). Flow cytometry has only been used to evaluate T lymphocyte proliferation in response to an anti-CD3 antibody or anti-CD3/CD28 coated beads, as well as B cells in response to anti-BCR plus CpG, which were found to be defective (65). However, patients with normal lymphoproliferative response have also been reported (131). There is no anti-CTPS1 fluorochrome-conjugated antibody commercially available. Therefore, CTPS1 expression is analyzed by western blot (65). CTPS1 expression by flow cytometry has been validated in HepG2 cells through incubation of primary unconjugated antibody followed by a dye-conjugated secondary antibody staining (153). This staining strategy represents a potential approach to screen patients with CTPS1 deficiency by flow cytometry.

#### ITK Deficiency

Mutations in the IL-2-inducible T cell kinase (ITK) causes a life-threatening syndrome of immune dysregulation and therapy-resistant EBV-associated lymphoproliferative disease (154–156). ITK is a signaling molecule located proximal to the TCR (Figure 4). ITK is expressed in thymocytes and peripheral T cells, regulating the thresholds of TCR signaling and specific development of CD8^+^ T cells (131). Flow cytometry analysis has shown that ITK deficient patients exhibit a reduced TCR-mediated calcium flux in T cells (67) and an absence of NKT cells as determined by the lack of TCR Vβ11 and TCR Vα24 double-positive cells (156).

#### MAGT1 Deficiency

In addition to its essential role as a co-factor for nucleic acids and metabolic enzymes (157, 158), a critical role of magnesium ion (Mg^2+^) in immune responses has been demonstrated by disease-causing mutations in the magnesium transporter 1 gene (*MAGT1*). Li et al. (68) reported Mg^2+^ as an intracellular second messenger following TCR activation in patients with an X-linked inborn error of immunity characterized by CD4^+^ T cell lymphopenia, severe chronic viral infections (e.g., EBV infection associated with lymphoproliferative disease or lymphoma), and defective T lymphocyte activation. Flow cytometry was used by the authors to characterize several immunological defects, but not the expression of MAGT1, which was investigated by Western blots. A reduced CD69 expression by CD4^+^ T cells after anti-CD3 stimulation was identified, while the response to phorbol 12-myristate 13-acetate (PMA) plus Ionomycin was normal, thus suggesting a specific defective TCR signaling that was confirmed by impaired NF-κB and NFAT nuclear translocation using confocal microscopy. Reduced levels of naïve CD4^+^ T cells (CD27^+^, CD45RO^−^) expressing CD31, a cell surface marker of naive TREC-rich T cells, suggest a diminished thymic output (159–161). Kinetic analysis by flow cytometry also revealed abrogation of TCR-induced Mg^2+^ influx, which can be detected by the Mg^2+^-specific fluorescent probe, MagFluo4 (68). Another immunologic feature of the disease is the impaired cytotoxic function of NK and CD8^+^ T cells. Chaigne-Delalande et al. (162) elegantly demonstrated that decreased intracellular free Mg^2+^ causes impaired expression of the natural killer activating receptor NKG2D in NK and CD8^+^ T cells, impairing cytolytic responses against EBV.

#### PRKCD Deficiency

Protein kinase C delta (PKCδ) (69–71, 163) belongs to a family of at least 11 serine/threonine kinase members involved in several pathological conditions (164, 165). Mutations in this gene cause a monogenic disease that presents either as SLE-like disease or as autoimmune lymphoproliferative syndrome (ALPS)-like disorder. PKCδ deficiency is associated with uncontrolled lymphoproliferation and chronic EBV infection. Immunologically, human PKCδ deficiency results in a B cell disorder characterized by B cell resistance to apoptosis, B cell hyperproliferation, increased production of autoantibodies, and decreased numbers of memory B cells (69–71, 163). A similar phenotype has been identified in PKCδ knockout mice (166–168), demonstrating the essential role of PKCδ in B cell tolerance. Flow cytometry applications to investigate this disease are designed to demonstrate increased B cell proliferation after anti-IgM stimulation, resistance to PMA-induced cell death (70), and the almost absence of CD27 expression on B cells (69), i.e., absence of memory cells.

#### X-Linked Lymphoproliferative Syndromes

X-linked lymphoproliferative syndrome (XLP) is a PID that presents with severe or fatal EBV infection, acquired hypogammaglobulinemia, malignant lymphoma, and HLH (72, 169). Most XLP cases are due to mutations in the SH2 domain protein 1A (*SH2D1A*) gene (XLP type 1), which encodes the signaling lymphocytic activation molecule (SLAM)-associated protein (SAP) (72). SAP is an adapter molecule that controls several signaling pathways involved in lymphocyte activation, proliferation, cytotoxicity, and also promotion of apoptosis [Figure 4; (170–172)]. The defect in antibody production exhibited by SH2D1A-deficient patients probably arise from impaired CD4^+^ T cell interaction with B cells rather than an intrinsic B cell failure (169, 173).

**Figure 6:**
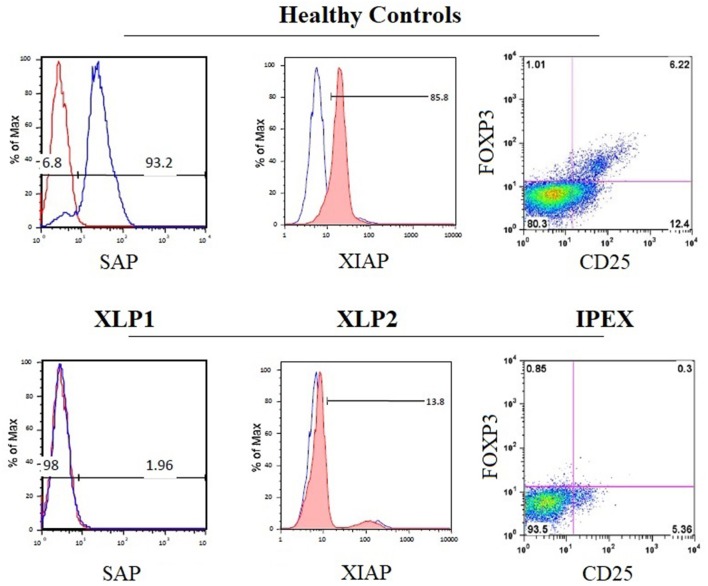
Disease-specific flow cytometry tests for PIDs with immune dysregulation. The histograms show the normal expression of SAP and XIAP from healthy control lymphocytes (upper panels) compared with the absent expression of SAP and XIAP in lymphocytes from patients with X-linked lymphoproliferative syndrome type 1 (XLP1) and XLP2 (lower panels), respectively. The dot plot exhibits the absence of nuclear forkhead box P3 (FOXP3) expression in CD4^+^CD25^+^ regulatory T cells from patient PBMCs with immune dysregulation, polyendocrinopathy, enteropathy, X-linked inheritance syndrome (IPEX) (bottom right panel) compared with healthy control PBMCs (top right panel).

Mutations in the gene encoding the X-linked inhibitor of apoptosis (*XIAP*), which inhibits caspase-3,−7, and−9 by direct binding (174), are responsible for XLP type 2 syndrome (73).The clinical phenotype and the disease pathogenesis have been reviewed and compared in detail elsewhere (129, 172, 175, 176). Flow cytometry can be used to evaluate apoptosis, in order to distinguish both XLP forms. Due to the distinct physiological roles of SH2D1A and XIAP, enhanced apoptosis of T lymphocytes is observed in patients with XIAP-deficiency, while the absence of SAP in SH2D1A deficiency is consistently associated with impaired cell apoptosis (133, 170, 172). This might explain why cytopenia is common in XIAP but not in SH2D1A deficiency (129). The EBV-associated immune dysregulation in XIAP deficiency might, in part, be due to the combination of an intrinsic exacerbated proliferation of immune cells plus the incapacity to respond to EBV. The lymphoproliferative disease reported in SH2D1A deficiency seems to be more the consequence of extrinsic and constant stimulation induced by EBV that cannot be properly controlled. For both XLP forms, flow cytometry to test intracellular testing for SAP and XIAP protein expression is available [Figure 6; (34)]. In addition, flow cytometric testing has demonstrated that the absence of SAP or XIAP proteins results in reduced numbers of circulating NKT (Vα24TCR^+^/Vβ11TCR^+^) cells (73).

## Syndromes With Autoimmunity

**Figure 7:**
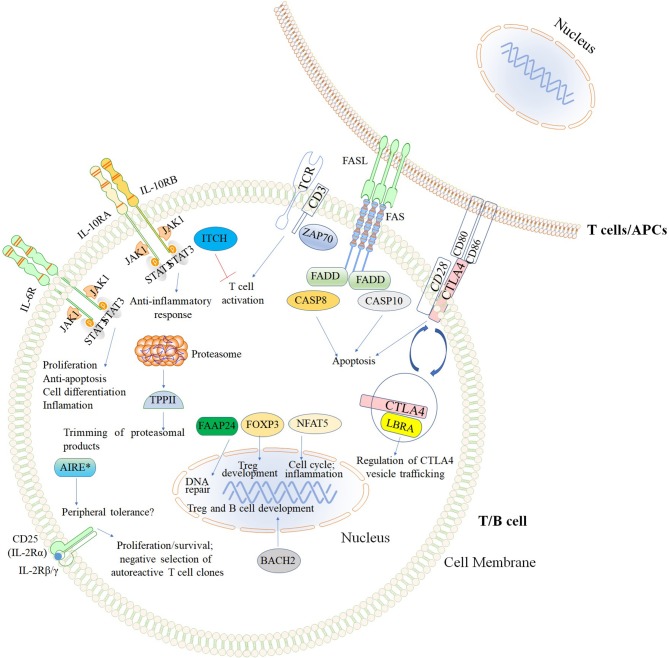
Inborn errors of immunity that cause Syndromes with autoimmunity and others. The illustration demonstrates the mutated genes associated with syndromes with autoimmunity, and are shown with different colored backgrounds. To allow a better comprehension of signaling pathways, other molecules (TCR, CD3, CD28, CD80, and CD86) not associated with syndromes associated with autoimmunity are shown in white background. Nomenclature as designated by the 2017 IUIS phenotypic classification of PIDs (24). The illustration was constructed using Motifolio Drawing Toolkits (48).

**Figure 8:**
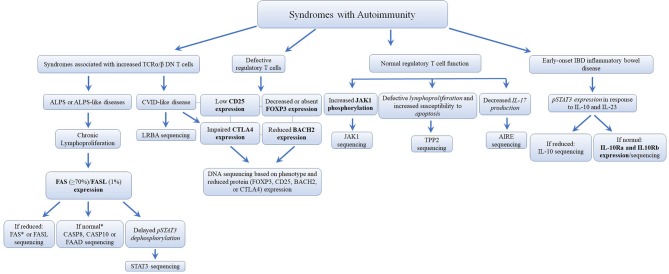
Flowchart depicting the recommended immunophenotypic analysis used to define the molecular genetic defects of patients with immunodeficiency syndromes with Autoimmunity. In those cases with normal protein by flow cytometry, if there is a strong clinical indication for a specific immune dysregulation disease, it is recommended to perform gene sequencing to exclude missense mutations that do not impair protein expression. It is estimated that mutations in the FAS receptor are the most frequent pathology of ALPS (≅70% of genetically defined ALPS) (177, 178). However, the frequency of other immune dysregulation syndromes remains unknown. Bold and italic texts are disease-specific and non-disease specific flow cytometry tests, respectively.

The second major group of diseases of immune dysregulation named “Syndromes with Autoimmunity and Others,” is subdivided based on the increased percentage of CD4^−^CD8^−^TCRα/β (double negative [DN] T cells), on Treg defects, and the development of colitis (24). The 21 disease-causing genes belonging to this group are represented in Figure 7 as well as a summarized guideline (Figure 8) which describes the flow cytometric assays required to diagnose patients with syndromes that include autoimmunity.

### Syndromes Associated With an Increased Percentage of CD4^−^CD8^−^TCRα/β Cells

#### Autoimmune Lymphoproliferative Syndromes (ALPS)

TCRα/β DN T cells are useful biomarkers, frequently elevated in children with autoimmune lymphoproliferative syndromes (ALPS) (179). The immunological functions of these cells have been reviewed in detail elsewhere (180). However, their precise role in the pathogenesis of autoimmune diseases is not well understood (179). ALPS is caused by mutations in five different genes: *FAS, FASL, FADD, CASP8, and CASP10*. The interaction between Fas (CD95) and Fas ligand or FasL (CD178), both expressed by activated T lymphocytes (the former also present on other cell types), triggers the formation of a death-inducing signaling complex (181, 182). This process involves the recruitment of Fas-associated death domain (FADD), cysteine-aspartic acid protease 8 (*CASP8*), and *CASP10*, initiating a cascade of signaling events that result in apoptotic cell death (183). This process regulates lymphocyte life span and promotes the elimination of autoreactive lymphocytes (Figure 7). The syndromes caused by mutations in these five genes have been classified by the National Institutes of Health (NIH) (177) as ALPS-FAS cell surface death receptor (the most frequent) (75), ALPS-FASL (76), ALPS-Caspase 8 (77), ALPS-Caspase 10 (78), and the FADD-deficiency (79). These disorders generally present as lymphadenopathy, splenomegaly, and autoimmune manifestations such as autoimmune hemolytic anemia, and severe recurrent thrombocytopenia (75–79, 184). Laboratory findings also include polyclonal hypergammaglobulinaemia, T lymphocyte apoptosis defect, and increased percentages of TCRα/β DN T cells (177).

Flow cytometry analysis demonstrates defective T cell apoptosis in response to anti-Fas antibody, recombinant FasL, or after phytohaemagglutinin (PHA)-/IL-2 stimulation by using FasT Kill assays or AV/PI or 7-AAD-staining. The technique of detecting increased percentages of TCRα/β DN T cells within peripheral blood mononuclear cells (PBMCs) is well established (76, 185, 186). Moreover, protein expression of FAS (187) and FASL (186) (both after T-cell blast generation by PHA plus IL-2) by flow cytometry is available to investigate ALPS-FAS and ALPS-FASL, respectively. Although the other ALPS forms (due to FADD (79), CASP8, or CASP10 deficiency) have not yet been studied by flow cytometry due to the unavailability of specific fluorescent conjugated antibodies, mutations in the FAS receptor is the most frequent disease form of ALPS found in ~70% of genetically defined ALPS (177, 178) thereby making flow cytometry an essential screening tool for patients suspected to have ALPS.

#### STAT3 Gain-of-function Mutations

While heterozygous germline inactivating mutations in the signal transducer and activator of transcription 3 (STAT3) with dominant negative effect cause autosomal dominant hyper IgE syndrome (188), heterozygous gain-of-function (GOF) mutations in STAT3 result in an ALPS-like phenotype (81). Patients can develop early-onset poly-autoimmunity (e.g., type 1 diabetes), autoimmune hypothyroidism, enteropathy, pulmonary disease, cytopenias, arthritis, short stature, myelodysplastic syndrome, aplastic anemia, and lymphocytic leukemia (81, 189, 190). Increased percentages of TCRαβ^+^-DN T cells are occasionally identified (189). So far, STAT3 GOF mutations have been shown to enhance transcriptional activity and delay dephosphorylation of STAT3, without inducing constitutive phosphorylation as shown by flow cytometry studies. In agreement with the involvement of STAT3 in the inhibition of Tregs (191, 192) and enhancement of Th17 cell differentiation (193), flow cytometry has also shown increased Th17 levels while the FOXP3^+^CD25^+^ Treg population is reduced and the expression of CD25 (IL2RA) is decreased in patients with STAT3 GOF mutations (189). Due to its activity as a repressor of FAS-FASL activity, decreased FASL-induced apoptosis has been observed (190).

#### LRBA Deficiency

Mutations in the lipopolysaccharide responsive beige-like anchor protein (*LRBA*) gene cause a common variable immunodeficiency (CVID)-like disease with predominant antibody deficiency (hypogammaglobulinemia) and autoimmunity (e.g., autoimmune hemolytic anemia as well as atrophic gastritis with autoantibodies against intrinsic factor, autoimmune enteropathy, hypothyroidism, myasthenia gravis, polyarthritis), and inflammatory bowel disease (80, 194, 195). The phenotype of LRBA deficiency has been well-characterized elsewhere (196). LRBA is highly expressed in immune cells such as T and B cells (80). The application of flow cytometry to screen patients with LRBA deficiency has been recently developed (197) as well as its application to evaluate several immunopathological mechanisms of this disease. More than 70% of the LRBA-deficient patients have reduced levels of Tregs (196) (CD4^+^CD25^+^FOXP3^+^), which may be related to the low surface expression of cytotoxic T lymphocyte–associated antigen 4 (CTLA4 or CD152) (198). CTLA4 is a cell surface molecule required for the proper suppressive function of Tregs (199–201). The reduced CTLA4 levels can be explained by the fact that LRBA is a regulator of CTLA4 vesicle trafficking [Figure 5; (197)]. Increased percentages of TCRα/β DN T cells have been found in up to 50% of LRBA-deficient patients (196). Several other defects associated with LRBA deficiency have been identified by flow cytometry (80). Among them are increased B cell apoptosis, low levels of IgG^+^IgA^+^CD27^+^ switched-memory B cells, reduced B cell proliferation, and impaired activation (as measured by CD138 expression).

### Defective Regulatory T Cells

The next two subgroups of Syndromes with Autoimmunity are based on the presence or absence of Treg defects (24). Tregs play a central role in peripheral immune tolerance, which controls the response of mature B and T cells that egressed from the primary lymphoid organs (202–204). Several autoimmune diseases have demonstrated the essential role of Tregs (202, 205), whose development is orchestrated by the transcription factor FOXP3.

#### Immune Dysregulation Due to Abnormal Tregs

##### IPEX

The immune dysregulation, polyendocrinopathy, enteropathy, X-linked syndrome (IPEX) is caused by loss of function mutations in the *FOXP3* gene (82). Clinical, immunological, and molecular features of IPEX syndrome have recently been characterized in a large cohort of patients (206). Flow cytometry of CD4^+^FOXP3^+^CD25^+^ cells is well established to screen patients suspected to have IPEX who normally have low or absent nuclear FOXP3 expression in Tregs [Figure 6; (34)]. However, patients with missense mutations in *FOXP3* may present with normal protein expression and are not suitable for flow analysis. The identification of *FOXP3* mutations is essential to differentiate patients with IPEX from those with IPEX-like syndromes caused by mutations in other immune regulatory genes (e.g., *LRBA, CTLA4*, and CD25) (83, 206).

##### CD25 deficiency

Although CD25-deficient patients display normal percentage of FOXP3^+^ cells, mutations in the *CD25* gene, which encodes the high-affinity subunit IL-2 receptor alpha chain (IL-12RA) of the tripartite receptor for IL-2 (83), causes an IPEX-like syndrome. This observation is explained by the fact that CD25, which can be detected by flow cytometry, is required for the production of the immunoregulatory cytokine IL-10 by Tregs (207). This suggests that CD25 is required for the function but not the survival of Tregs (207). CD4^+^ lymphocytes are decreased in numbers, and the proliferative response following stimulation with anti-CD3, PHA, or other mitogens is diminished (208). In addition, CD25 deficiency decreases apoptosis in the thymus, impairing negative selection of autoreactive T cell clones, resulting in inflammation in multiple organs (208).

Flow cytometry has also defined a role of CD25 in NK cell maturation and function, as suggested by the accumulation of CD56^bright^CD16^high^ and reduced frequency of CD56^dim^CD16^hi^ NK cell in the peripheral blood as well as the expression of higher amounts of perforin and granzyme B. Increased degranulation (by increased CD107a expression) while reduced IFN-γ production by NK cells has also been reported (209).

##### CTLA4 deficiency

Mutations in the inhibitory receptor *CTLA4*, which acts to terminate the proliferation of activated T cells, have recently been recognized as a monogenic cause of CVID (210, 211). Therefore, for diagnostic assays of CTLA4, LRBA, and BACH2, defects in these molecules need to be evaluated in parallel (Figure 7; see section BACH2 Deficiency). CTLA4 is also constitutively expressed by Tregs and functions as a key checkpoint molecule for immune tolerance (211, 212). Details of CTLA4 biology and immunophenotyping of CTLA4 haploinsufficiency have recently been reviewed (213, 214). Briefly, CTLA4 competes effectively with CD28 because of higher affinity for binding to the costimulatory molecules CD80 and CD86, which are expressed on the surface of antigen-presenting cells (215). Patients with CTLA4 haploinsufficiency develop a T cell hyperproliferative syndrome resulting in lymphocytic infiltration of multiple organs (e.g., brain, gastrointestinal, and lung), autoimmune thrombocytopenia, hemolytic anemia, and other cytopenias, as well as hypogammaglobulinemia (84, 210), and increased susceptibility for cancer (216). Decreased CTLA4 expression can be demonstrated by flow cytometry. This tool is also useful to assess the effect of different mutations on CTLA4 function, which would normally require complex assays. For instance, flow cytometry can be used to demonstrate that CTLA4 loses its ability to interact with its natural ligands (CD80 and CD86), to traffic from the intracellular compartment to the cell membrane, and to inhibit T cell activation by physical removal of CD80/CD86 by CTLA4-mediated trans-endocytosis (211, 217, 218).

##### BACH2 deficiency

The gene encoding the BTB and CNC homology 1, basic leucine zipper transcription factor 2 (BACH2) is involved in the maturation of T and B lymphocytes. BACH2 is required for class switch recombination (CSR), somatic hypermutation (SHM) of immunoglobulin genes, and generation of regulatory T cells (219, 220). BACH2 haploinsufficiency has recently been associated with CVID and lymphocytic colitis. Low BACH2 protein expression in CD4^+^, CD8^+^ T and B lymphocytes can be demonstrated by flow cytometry, together with significantly decreased numbers of Foxp3^+^ Treg cells, increased Th1 cells, reduced CD19^+^CD27^+^ memory, and low IgG class-switched CD27^+^IgG^+^ B cells (85).

#### Normal Treg Function

##### APECED

The discovery that mutations in the autoimmune regulator (*AIRE*) gene cause the autoimmune-polyendocrinopathy-candidiasis-ectodermal-dystrophy (APECED) syndrome (221) provided the novel concept that a monogenic defect can cause a systemic human autoimmune disease (86). The endocrinopathies presented by APECED patients are characterized by hypoparathyroidism, hypothyroidism, adrenal failure, gonadal failure, and autoimmune hepatitis. The ectodermal dystrophies comprise vitiligo, alopecia, keratopathy, and dystrophy of dental enamel, nails, and tympanic membranes (86, 222).

AIRE mediates central T cell tolerance by promoting the expression of thousands of tissue-specific self-antigens by medullary thymic epithelial cells (mTEC), leading to the deletion of T cells with strongly self-reactive TCR (223). Extrathymic AIRE expression has recently been reported in response to antigen and interleukin 2 stimulation in human peripheral blood cells such as CD4^+^ T cells, suggesting a role of AIRE in mature lymphocytes (224). However, there is no flow cytometry assay available to analyze AIRE expression in peripheral blood lymphocytes. To explore the expression of AIRE in CD4^+^ T cells to screen patients with APECED could improve the precise diagnosis of this disease, once the screening is currently based on the presence of the classical triad of CMC, hypoparathyroidism and adrenal insufficiency (Addison's disease) (225).

##### Tripeptidyl-peptidase II deficiency

Tripeptidyl peptidase II (TPPII) is a cytosolic peptidase that works downstream of proteasomes in cytosolic proteolysis by trimming proteasomal degradation products [Figure 7; (226)]. TPPII modulates several cellular processes, including antigen presentation by major histocompatibility complex (MHC) I molecules, T cell proliferation, and survival (87, 227). Among others, patients with TPPII deficiency develop autoimmune manifestations (e.g., immune hemolytic anemia, immune thrombocytopenia, and other cytopenias), and they are susceptible to viral infections such as CMV and severe chickenpox (87).

Although not used to assess TPPII expression in lymphocytes for establishing the diagnosis of TPPII deficiency, flow cytometry has been broadly employed to immunophenotypes and characterize lymphocyte function in affected patients. Lymphocytes from TPPII-deficient patients express higher levels of HLA class I molecules, present a skewed T-effector memory phenotype, and have a predominant CD8^+^CD27^−^CD28^−^CD127^−^ phenotype (87), which has been associated with enhanced effector functions and increased percentages of IFN-γ- and IL-17- positive T cells, as well as high levels of T-bet and perforin expression. Defective lymphoproliferation and increased susceptibility to apoptosis were also characterized by flow cytometry using Carboxyfluorescein succinimidyl ester (CFSE) and AV/PI. Furthermore, the patients showed increased levels of CD21^low^ cells, an autoreactive B cell population often associated with CVID and autoimmune diseases. CD21^low^ B cells are thought to have undergone activation and proliferation *in vivo* while exhibiting defective proliferation in response to B cell receptor stimulation (228, 229).

##### JAK1 gain-of function

The janus kinase 1 (JAK1) plays a central role in cytokine (e.g., interferon-α, IFN-γ, IL-6) signaling by phosphorylating STAT proteins (e.g., STAT1, STAT2, and STAT3). STAT proteins translocate to the nucleus and activate the transcription of many genes involved in immune responses (230). A family with a JAK1 germline GOF mutation that causes a systemic immune dysregulatory disease has recently been reported. Affected patients present with severe atopic dermatitis, profound eosinophilia, and autoimmune thyroid disease. A phospho-flow cytometry assay was able to demonstrate increased JAK1 and STAT1 phosphorylation at baseline and following IFN-α stimulation as well as enhanced IL-6-induced STAT3 phosphorylation (88).

##### Challenges to evaluating Treg function by flow cytometry

Due to their relevant pathophysiological role in the maintenance of immune homeostasis, we briefly reflect on the challenges associated with evaluating Treg number and function by flow cytometry. Distinct markers have been used to characterize human CD4^+^ regulatory T cells since their first *ex-vivo* characterization in 2001 (231–233). The stable expression of the transcription factor FOXP3 represents one of the hallmarks of Tregs in both human and mice (234) and has been used to evaluate Tregs by flow cytometry, not only in PIDs with immune dysregulation but also other human diseases, including cancer (235) and diabetes (236). However, the functional characterization of human Tregs by flow cytometry still represents a challenge due to several factors; (I) FOXP3 can also be transiently expressed by activated CD4^+^ T cells (237, 238); (II) FOXP3 evaluation requires the permeabilization of the nucleus membrane thereby impeding the possibility of FACS-sorting; (III) Circulating Tregs represent a very low frequency of the blood composition (representing 10% of the CD4^+^ T cell compartment) and therefore a large number of PBMCs are required for adequate analysis. (IV) Classic Treg definition requires the *ex-vivo* evaluation of their suppressive capability.

Phenotypically, the evaluation of Tregs goes beyond the expression of FOXP3 in CD4^+^ T cells, requiring the combination of distinct surface markers. In order to detect the high expression of the alpha chain of the IL-2 receptor (CD25) (232, 233), flow cytometric panels have shown that Treg cells exhibit low expression of both CD45RA (239) and IL-7 alpha receptor (CD127) (240, 241). Recent works have also shown that Tregs from tissues might express high levels of activation markers such as the coinhibitory receptor T cell Ig and ITIM domain (TIGIT) (242), the inducible T-cell co-stimulator (ICOS) (243), and the ectonucleotidase CD39 (244–246), which could be used for further *ex-vivo* isolation and characterization.

Another challenge for the laboratorial evaluation of Tregs consist of the low frequency of these cells in peripheral blood, which limits adequate functional assessment of these cells. To overcome this limitation, *in vitro* strategies for Treg expansion may include an initial cell enrichment step by selecting T cells, phenotypically characterized by CD4^+^CD25^high^CD127^low^ expression, that will subsequently be subjected to cell culture in the presence of IL-2, rapamycin or TCR-stimulation (e.g., anti-CD3 or APCs) (247–249). These strategies may be considered to achieve the number of cells required for screening or classical suppression assays using cells from patients with PIDs and immune dysregulation. In this context, Tregs are co-cultured and proliferated with conventional CD4^+^ T cells or CD8^+^ T cells under polyclonal stimulation followed by assessing suppression of proliferation with fluorescent-labeling methods. The ratios of Tregs to target cells, duration of co-culture and readout need to be adapted to each set of assays, considering variation of donors, cell viability and the sensitivity of the suppression method (250).

### Immune Dysregulation With Early Onset Colitis

#### IL-10, IL-10Ra, and IL-10Rb Deficiencies

**Figure 9:**
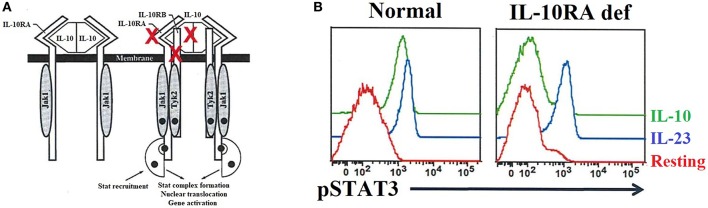
IL-10 and IL-10 receptor deficiencies and the relevant role of flow cytometry analysis for precise diagnosis. **(A)** The interaction between IL-10 and its receptor (left side) as well as the downstream signaling events (right side) are exhibited. The red X highlights the IL-10 and IL-10 receptors (IL-10RA/B) that if mutated cause immune dysregulation with early onset colitis. **(B)** Flow cytometric histograms from a healthy control and an IL-10RA deficient patient, in resting or after IL-23 or IL-10 stimulation, illustrating the importance of flow cytometric application for functional assessment of inborn errors of immunity.

Interleukin 10 (*IL-10*) is an important anti-inflammatory cytokine produced by cells like APCs. Early-onset (within the first months of life) of severe inflammatory bowel disease (EO-IBD), i.e., Crohn's disease and ulcerative colitis (UC), can be caused by IL-10 and IL10- receptor deficiencies (89, 90, 251). The expression of both IL-10 receptor alpha (IL-*10RA*) and IL-10 receptor beta (*IL10RB*) can be assessed by flow cytometry (90). Of note, IL-10 binds to its receptor, leading to the activation of the JAK1-STAT3 pathway [Figure 9A; (252)]. Normal or defective IL-10-induced phosphorylation of STAT3 in T cells has been evaluated by flow cytometry to distinguish patients with EO-IBD due to IL-10 or IL-10R deficiencies (Figure 9B). Recombinant IL-6 or IL-23 are used in parallel with IL-10 as stimuli to distinguish the specificity of IL-10 or IL10R deficiencies.

## Conclusion and Future Perspectives

Since the identification of the specific mutation is the definitive approach for a specific molecular diagnosis, flow cytometry represents an extremely useful and versatile tool to effectively and rapidly evaluate patients with PIDs at relatively low costs (32–35). Of note, most of the other PIDs associated with immune dysregulation (Figure 3) seem to be rare diseases. This current landscape is also influenced by the fact that, while some diseases have been described earlier (e.g., mutations in *FAS, FASL*, and *LYST*) (75, 184) and investigated in more detail, the molecular defects that cause most PIDs with immune dysregulation have only recently been discovered (Figure 1). However, we can confidently estimate that *PRF1 deficiency* accounts for 30–50%, and UNC13D deficiency for up to 20% of all FHL cases (34, 105, 108, 123), and mutations in the FAS receptor are the most frequent cause of ALPS [~70% of genetically defined ALPS (177, 178)]. The incidence of several other PIDs with immune dysregulation remains to be determined when additional patients are discovered. While more than 250 patients with Chediak–Higashi syndrome due to LYST deficiency were described 13 years ago (**?**), other PIDs we included in this review have been reported only in the last decade and we expect that only a small proportion of these patients have been discovered to date. The establishment of more laboratories capable of molecularly characterizing PIDs with immune dysregulation syndromes throughout the world, including developing countries, will be essential in advancing this new field of immunology. This will allow us to elucidate which defects are indeed rare or common.

Since these syndromes are rare, there is not a high request of specialized assays (e.g., FAS/FASL expression) when compared to other less specialized laboratory tests (e.g., complete blood count and quantitative immunoglobulins determination). Consequently, while the former assays are routinely only performed in PID research centers (often in state universities), which are supported by research grants, less specialized examinations are broadly available in most laboratories. We hope that improving the diagnoses of previously described and newly discovered PIDs with immune dysregulation will encourage governments and other funding sources to promote the establishment of new PID specialized laboratories in underserved geographic areas such as developing countries, where the true incidence of PIDs with immune dysregulation remains to be determined.

Finally, beyond its utility as a screening tool for patients with symptoms of immune dysregulation, flow cytometry has helped to characterize novel immunopathological mechanisms of several recently reported new PIDs. However, new flow cytometric technologies such as time-of-flight mass cytometry (CyTOF) (253) have not yet been applied for characterizing the immunopathology of immune dysregulation syndromes. Equally, flow cytometry is not currently applied in the context of systems immunology studies (254, 255) to better understand the immunopathology of diseases of immune dysregulation. For instance, traditional flow cytometry can be used to validate the findings obtained from combinatorial techniques such as CyTOF with high-throughput sequencing of mRNA (RNA-seq) or mass spectrometry, and uncovering systemic immunology defects (256, 257). Systems immunology will provide a more comprehensive understanding of the role of specific molecules across immune cells, potentially revealing novel therapeutic targets for patients with diseases of immune dysregulation.

## Author Contributions

OC-M: acquisition of data, wrote the manuscript, edited the manuscript, and proof reading. LS, NE, RR, and GS: wrote the manuscript. EO: figure configuration and wrote the manuscript. BA-R: wrote the manuscript, proof reading, and edited the manuscript. HO and AC-N: proof reading, wrote, and edited the manuscript.

### Conflict of Interest

EO was employed by company Immunogenic Inc., Brazil. The remaining authors declare that the research was conducted in the absence of any commercial or financial relationships that could be construed as a potential conflict of interest.
